# Retraction: Carbapenem resistance and *Acinetobacter baumannii* in Senegal: The paradigm of a common phenomenon in natural reservoirs

**DOI:** 10.1371/journal.pone.0324349

**Published:** 2025-05-09

**Authors:** The PLOS One Editors

The *PLOS One* Editors retract this article [1,2] due to concerns about compliance with the PLOS Human Subjects Research policy.

The Methods section in [1] reports that the study involved the collection of head lice from women with no clinical symptoms from different locations in Senegal from October 2010 to January 2011. In addition, the study reports the inclusion of stool samples collected from healthy individuals in Dielmo and Ndiop (Senegal) between 2008–2010. The ethics approval reported in the article refers to agreement #11–010 issued by the Ethic Committee of the Institute Fédératif de Recherche 48 (IFR48), Marseilles, France. The article does not report prospective, local ethics approval from an institution or agency in Senegal. Furthermore, PLOS noted that the ethics approval number #11–010 was also reported in [3], despite there appearing to be major differences in the locations and populations described in these studies.

A representative of the Aix-Marseille Université Ethics Committee stated that the study complied with applicable legislation and ethical standards. They stated that the samples collected in this study are considered to be human waste, and that the Aix-Marseille Université Ethics Committee concluded that the study did not require ethics approval from a Comité de Protection des Personnes according to French law. The representative provided a copy of the French ethics approval document #11–010 issued by the IFR48 and copies of the Senegalese ethics approval documentation N° 969 MSP/DS/DER and N° 00.86 MSP/DS/CNERS. They commented that the #11–010 document grants approval for the secondary analysis of previously collected samples, as opposed to approval for the collection of the samples.

PLOS reviewed the documentation provided by the institution and concluded that the documents did not resolve the concerns. Specifically,

- The French ethics approval document #11–010 specified that samples were not collected specifically for this study, samples were anonymized, and patients provided consent. However, it does not mention approval for the secondary analysis of the lice samples and the approval is dated September 14, 2011, after the sample collection dates reported in the article. The *PLOS One* policies for Human Subjects Research states that studies involving human participants must obtain prior ethics approval, and PLOS has not received any evidence to confirm that the participants provided prior consent for the secondary analysis of their samples as part of a separate study.
- The Senegalese ethics approval document N° 969 MSPM/DS/DER was issued on September 25, 2006 for a study titled, «*Etude de l’histoire naturelle du paludisme: protocole d’étude mené dans les villages de Dielmo et Ndiop arrondissement de Toubacouta Région de Fatick/Senegal* ». The study described in the document does not appear to match the study reported in [1]. The document does not mention approval for the secondary analysis of the collected samples as part of a separate study.
- The Senegalese ethics approval document N° 00.86 MSP/DS/CNERS was issued on June 02, 2010 for a study titled “*Identification des agents pathogènes responsables de fièvre au Sénégal. Réalisation de tests diagnostiques chez les maladies consultant dans les dispensaires de Dielmo at Ndiop”.* It does not provide prospective approval for the collection of stool samples between 2008–2010. Furthermore, it specifies approval for carrying out diagnostic tests on patients, whereas the *PLOS One* article [1] reports collection of samples from healthy individuals without clinical symptoms. In addition, the document does not mention approval for the secondary analysis of the collected samples as part of a separate study.
- The *PLOS One* article [1] reports that the majority of samples were collected from children; the ages of the children were not reported and the article does not report information about informed consent and assent considerations for minors.

All authors either did not respond directly or could not be reached.
